# Elucidation of Dithiol-yne Comb Polymer Architectures by Tandem Mass Spectrometry and Ion Mobility Techniques

**DOI:** 10.3390/polym16121665

**Published:** 2024-06-12

**Authors:** Kayla Williams-Pavlantos, Abdol Hadi Mokarizadeh, Brennan J. Curole, Scott M. Grayson, Mesfin Tsige, Chrys Wesdemiotis

**Affiliations:** 1 Department of Chemistry, University of Akron, Akron, OH 44325, USA; knw55@uakron.edu; 2 School of Polymer Science and Polymer Engineering, University of Akron, Akron, OH 44325, USA; am555@uakron.edu (A.H.M.); mtsige@uakron.edu (M.T.); 3 Department of Chemistry, Tulane University, New Orleans, LA 70118, USA; bcurole1@tulane.edu

**Keywords:** comb polymers, tandem mass spectrometry, ion mobility, molecular modeling

## Abstract

Polymers have a wide range of applications depending on their composition, size, and architecture. Varying any of these three characteristics can greatly impact the resulting chemical, physical, and mechanical properties. While many techniques are available to determine polymer composition and size, determining the exact polymer architecture is more challenging. Herein, tandem mass spectrometry (MS/MS) and ion mobility mass spectrometry (IM-MS) methods are utilized to derive crucial architectural information about dithiol-yne comb polymers. Based on their unique fragmentation products and IM drift times, dithiol-yne oligomers with distinct architectures were successfully differentiated and characterized. Additionally, experimental collision cross-sections (Ω) derived via IM-MS were compared to theoretically extracted Ω values from molecular dynamics simulated structures to deduce the architectural motif of these comb oligomers. Overall, this work demonstrates the benefits of combining various mass spectrometry techniques in order to gain a complete understanding of a complex polymer mixture.

## 1. Introduction

Polymeric materials play an important role in numerous medical, consumer, and industrial applications. The utility of a polymeric product strongly depends on its macroscopic properties, which are largely determined by the polymer’s chemical characteristics, including its elemental composition, overall size (molecular weight), and molecular architecture. Several studies have documented that similar polymer sizes and chemistries but differing macromolecular architectures (linear, cyclic, branched, star, crosslinked) can result in unique physical [1], mechanical [2,3,4], electrical [5], and optical properties [6].

Branched polymer architectures are defined by their primary backbone and secondary side chains. Star, comb, and bottlebrush polymers are all subclasses of the branched polymer family, varying in their connectivity between the backbone and side chains, as well as in their overall branching density. Tailoring the branch length and branching density has been shown to have a significant impact on the mechanical properties of the polymer [7,8]. Specifically, having a higher degree of branching density leads to less flexible polymers with lower melting points, while altering side chain length impacts overall entanglement and polymer rigidity [7]. As a result of these factors, designing branched polymers with specific branch lengths and density is of particular importance to the polymer community. Of recent interest has been the synthesis of controlled comb polymers with uniform branch spacing [9]. This, however, is often difficult to achieve and requires investing significant time and expenses.

**Figure 1:**
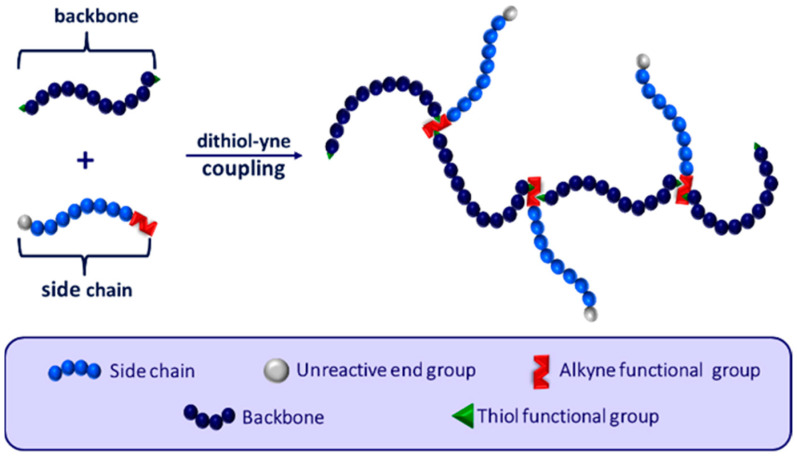
General schematic of the reaction between telechelic dithiol backbone units and monoalkyne side chains to form branching controlled comb copolymers. Adapted from ref. [13].

Comb polymers can be synthesized using *grafting from*, *grafting to*, or *grafting through* techniques [10,11,12]. Curole et al. recently developed a novel grafting through method that produces comb copolymers with oligomeric side chains at rapid speeds [9]. Branching is controlled by reacting a singly alkyne-terminated side chain and a telechelic dithiol macromonomer to form the copolymer backbone (Figure 1) [13]. This reaction proceeds via step-growth polymerization, where each alkyne can react with two thiol groups, resulting in a branched copolymer with controlled branch spacing. Further tailoring of the polymer properties is possible with this synthetic method by altering both the length and chemistry of the backbone macromonomer or side chain. 

Even with controlled synthetic methods, characterization of the molecular weight (MW), dispersity (PDI), purity, and architecture of the polymer is necessary before the product can be utilized in its designated application. Thus, it is crucial to employ advanced analytical methods that can accurately and efficiently determine the structural features of these materials. Gel permeation chromatography (GPC) is one of the most widely used characterization methods for polymeric materials. While beneficial for determining both polymer size and dispersity, it lacks the resolution to determine oligomeric and end group information [14,15]. Nuclear magnetic resonance (NMR) spectroscopy is useful to elucidate functional groups and polymer sequences but often suffers from bulk suppression, making it difficult to interpret and identify end groups [16]. As a result, mass spectrometry (MS) has become one of the primary methods for analyzing polymer systems [17,18,19,20]. The ability of single-stage MS to separate ions by their unique mass-to-charge ratio (*m*/*z*) allows for the distinction of repeat unit mass, end group mass, and molecular weight [17,18,19,20]. However, in some complex cases, single-stage MS techniques are not sufficient for complete polymer characterization. These cases pose the need for comprehensive multidimensional MS methods such as tandem MS and ion mobility MS [20]. 

Tandem mass spectrometry (MS/MS) is a valuable technique for investigating polymer sequences and architectures based on the unique fragmentation patterns of the selected macromolecular precursor ions. Polymers usually dissociate by either charge-induced or charge-remote pathways, resulting in characteristic backbone cleavages [20,21]. Over the last decade, numerous studies have applied MS/MS methods to characterize polymer and copolymer sequences [20,21,22,23], differentiate cyclic from tadpole and linear architectures [24,25], and analyze polymer topologies [20,26]. The fragmentation patterns presented in these publications have provided insight into common polymer fragmentation mechanisms that can be used to interpret the MS/MS spectra of new and more complex samples, such as those studied in the present work. 

In addition to MS/MS, ion mobility mass spectrometry (IM-MS) can be particularly beneficial for characterizing large macromolecules such as proteins [27,28,29,30] and synthetic polymers [20,31]. The unique ability of IM-MS to disperse ions differing in charge state and architecture opens the door for analyzing complex polymeric systems not amenable to other separation or spectroscopic techniques.

An important advantage of IM-MS is the ability to separate isomers and isobars. This is achieved by allowing the ions to travel through a pressurized electric field. The mobility or drift time of an ion through the ion mobility chamber correlates to its mass, charge, and collision cross-section (Ω), which is diagnostic of the ion’s size, shape, and architecture [32]. Coupling the ion mobility dimension with mass spectrometry allows for the observation and isolation of isomers with the same *m*/*z* ratio but differing molecular architectures as defined by their unique Ω values, which are obtained from the measured ion drift times or ion mobilities. In addition to these experimental results, molecular modeling is often used to determine the theoretical Ω value of each potential architecture. Comparison of the theoretical and experimental Ω values allows for a confident structural assignment of polymer architecture, protein folding, and molecular packing arrangement [33,34,35,36].

In this work, ESI-MS/MS and ESI-IM-MS are employed to characterize a complex mixture of dithiol-yne comb copolymers [13]. The ESI-MS/MS results provided insight into the various fragmentation patterns arising from low-molecular-weight dithiol-yne oligomers and suggested the formation of macrocycles. Further studies were carried out using IM-MS methods to distinguish isomeric linear and cyclic architectures. In conjunction with molecular modeling, the IM-MS experiments provided supporting evidence that the low-molecular-weight dithiol-yne oligomers were composed of macrocyclic chains [13].

## 2. Materials and Methods

### 2.1. Materials 

MeO-PEG_5_-OH was acquired from JenKem Technology USA (Plano, TX, USA). Propargyl bromide 97% (80% *w*/*w* in toluene stabilized with MgO) was acquired from Alfa Aesar (Haverhill, MA, USA). Sodium hydride 60% dispersion in mineral oil was acquired from Acros Organics (Waltham, MA, USA). 2,2-Dimethoxy-2-phenyl-acetophenone 99% (DMPA), 1,4-butanedithiol 97% (BDT), and poly-DL-alanine (MW 1000–5000) for the calibration of the ion mobility cell were acquired from Sigma-Aldrich (St. Louis, MO, USA). All solvents used for MS analysis were acquired from Fisher Scientific (Hampton, NH, USA). 

### 2.2. Synthesis of MeO-PEG_5_-Propargyl 

MeO-PEG_5_-OH (2.500 g, 9.9 mmol) was added to a dry two-neck 50 mL round-bottom flask. The PEG was heated to 60 °C in a water bath while stirring under vacuum to remove excess moisture. The flask was cooled, and dry tetrahydrofuran (25 mL) was transferred into the reaction flask using a cannula. Sodium hydride (476 mg, 19.8 mmol) and propargyl bromide (3.5 mL, 39.7 mmol) were added to the reaction flask and stirred under argon gas overnight. The reaction was quenched with isopropyl alcohol, and the precipitated salts were removed via gravity filtration. The filtrate was removed under reduced pressure, and the product was dissolved in DI water (20 mL) and washed with diethyl ether (10 mL × 3) to remove excess propargyl bromide. MeO-PEG_5_-propargyl was extracted from the water via a separatory funnel using chloroform (50 mL × 3) and a saturated sodium chloride brine solution (10 mL, ~6 M), dried with magnesium sulfate, and filtered. The solvent was removed under reduced pressure to afford a yellowish-orange liquid. (2.093 g, 83.7%) 

### 2.3. Synthesis of CP MeO-PEG_5_-BDT 

MeO-PEG_5_-Propargyl (0.104 g, 0.36 mmol) and BDT (84.1 µL, 0.72 mmol) were added to a 1.5-dram (5.5 mL) glass scintillation vial and solubilized in methanol (1.37 mL). A rubber septum was added, and the reaction flask was purged with argon gas for five minutes. A stock solution of DMPA was made in methanol at a 100 mg/mL concentration. The DMPA solution (0.06 mL, 3 wt. %) was injected into the reaction flask, and the solution was irradiated using a USpicy MACARON USND-3601 Professional UV Gel Lamp Nail Dryer (4 × 9 W 365 nm UV bulbs; Sunvalley Group, Shenzhen, Guangdong, China) for 30 min. The solvent was removed under reduced pressure, and the product was dissolved in DI water (10 mL) and washed with diethyl ether (10 mL × 3) to remove unreacted BDT and ethyl acetate (10 mL × 1) to remove the DMPA. The crude product MeO-PEG_5_-BDT was extracted from the water via a separatory funnel using chloroform (50 mL × 3) and a saturated sodium chloride brine solution (~6 M; 10 mL), dried with magnesium sulfate, and filtered. The solvent was removed under reduced pressure to afford a yellowish-orange semi-solid (0.130 g, 88.4%).

### 2.4. MS Instrument Conditions

All experiments were carried out on a Waters Synapt HDMS Quadrupole/Time-of-Flight (Q/ToF) mass spectrometer (Waters, Beverly, MA, USA) equipped with an ESI source and a traveling-wave ion mobility (TWIM) region between the Q and ToF mass analyzers composed of three compartments in the order of trap cell, TWIM cell, and transfer cell. The trap and transfer cells can be used in MS/MS experiments, while the TWIM cell is employed in IM-MS experiments.

Samples were initially dissolved in isopropyl alcohol (IPA) at 1.0 mg/mL. These solutions were then diluted to 30 ng/mL in a 90:10 IPA/methanol mixture before injection into the instrument. The ESI capillary voltage was set to 2.8 kV, the sampling cone voltage was set to 65 V, and the extraction cone voltage was set to 2.5 V. The desolvation gas was set to a flow rate of 600 L/h at a temperature of 350 °C, and the source temperature was set to 100 °C. All analyses were performed in positive ionization mode, and data interpretation was conducted using Waters’ MassLynx (version 4.1) and DriftScope (version 2.8) software. 

For ESI-MS/MS experiments, the trap collision energy (CE) was increased to 80–110 eV, depending on the precursor ion selected, while the transfer (CE) was set to 6 eV; both these cells were pressurized with Argon. All other instrument conditions were kept as described above. For ESI-IM-MS experiments, the trap and transfer CE were set to 6 eV and 4 eV, respectively; on the TWIM cell, the traveling wave height was set to 10 V, the traveling wave velocity to 300 m/s, and the IM buffer gas (N_2_) flow rate and pressure were set to 22.70 mL/min and 0.494 mbar, respectively.

### 2.5. Experimental Collision Cross-Sections

Experimental collision cross-sections (Ω) were derived from the corresponding IM-MS drift times, as has been described in detail elsewhere [37]. Calibration of the drift time scale was carried out by analyzing poly(alanine) standards at the same IM-MS conditions as the polymer samples in triplicate. Using the drift times of the singly and doubly charged poly(alanine) oligomers and their known collision cross-sections in He buffer gas (Ω_He_), calibration curves were constructed (Table S1 and Figure S1), which were used to obtain the experimental collision cross-sections (Ω_exp_) reported in this study [37,38]. Based on the proposed nomenclature for the presentation of IM-MS results [39], our experimental collision cross-sections are ^TW^Ω_N2_→_He_ values; in this acronym, the TW superscript designates the ion mobility variant (traveling wave), and the N_2_→He subscript specifies the buffer gas in which the drift times were measured (N_2_), and the buffer gas in which the calibrant collision cross-sections were obtained (He) [39]. This procedure renders Ω_exp_ values that can be compared with theoretically predicted collision cross-sections (Ω_theo_) in He buffer gas. 

### 2.6. Computational Modeling and Theoretical Collision Cross-Section Calculations

Theoretical modeling via annealing molecular dynamics calculations in a vacuum was performed using the Forcite module of Materials Studio 6.0 software [40] to better understand the dithiol-yne comb polymer molecular conformations. Intra- and inter-molecular interactions were defined using the COMPASS II forcefield [41], with the cut-off distance for both the van der Waals and electrostatic interactions set to 15.5 Å. Fluoride anions were used to neutralize the sodium cation in the system. The fluoride anion was placed in a fixed position at distances far from the molecule/sodium cation complex (>100 Å). Each structure was subjected to 50 annealing cycles, starting with 50 K as the initial temperature until it reached 1000 K at the midcycle. The number of heating ramps per cycle was set to 20 with 5000 steps per ramp. A step time of 1 femtosecond was used, and the most stable conformation of each cycle was generated into a trajectory file for further post-analysis. 

Theoretical Ω values were calculated using the MOBCAL program and He as collision gas [42]. For each comb copolymer, the corresponding x, y, and z coordinates of each atom, including the sodium counter ion, were copied into the software, and the calculations were run for 50 conformations. For each of the resulting candidate structures, the MOBCAL software provided three average cross-sections based on the projection approximation, electron hard sphere, and trajectory method [43]. Only the trajectory method values were considered as they provide the most accurate Ω values for the small oligomeric comb molecules studied in this work. This process was repeated for all 50 candidate structures from each oligomer, and the average Ω value (and std. deviation) calculated for each oligomer is reported as the corresponding Ω_theo_ (Table S2). 

## 3. Results and Discussion

### 3.1. ESI-MS Analysis

**Scheme 1:**
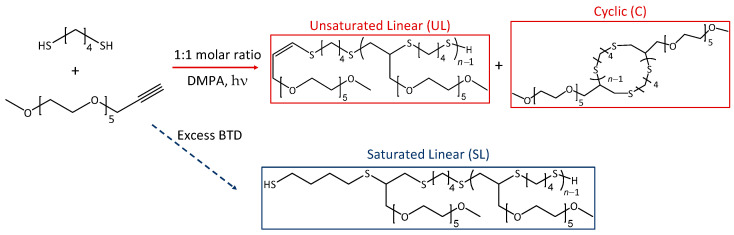
Synthetic scheme for the formation of dithiol-yne comb copolymers from 1,4-butanedithiol (BTD) and MeO-PEG_5_-propargyl using DMPA for initiation. The copolymer repeat unit with these reactants is C_18_H_36_O_6_S_2_ (412.195 Da monoisotopic mass).

The *grafting through* method, using photoinitiation of dithiol linkers and alkyne terminated poly(ethylene glycol) (PEG) side chains, has been shown to produce macromolecular dithiol-yne comb polymers [9]. When the dithiol and alkyne react in a 1:1 molar ratio, the resulting polymer retains a terminal double bond at the anti-Markovnikov position; this results in the formation of an unsaturated linear chain, which could undergo intramolecular cyclization to form a macrocyclic poly(thioether) chain decorated with PEG tails. However, if an excess of dithiol is used, an additional saturated linear product is formed via thiol addition at the terminal double bond, cf. Scheme 1.

Previous characterization of these combs using gel permeation chromatography (GPC) and proton nuclear magnetic resonance (^1^H NMR) provided insight into the molecular weight and oligomeric composition of the product. The average molecular weight (*M*_n_) with 1:1 molar equivalents of BDT to MeO-PEG_5_-propargyl, calculated by ^1^H NMR experiments based on the ratio of the end groups to the repeating units, was significantly higher than that calculated via GPC, suggesting that chain cyclization had occurred [9]. This preliminary estimation, however, requires further analysis to confirm the microstructure of the unsaturated product.

**Figure 2:**
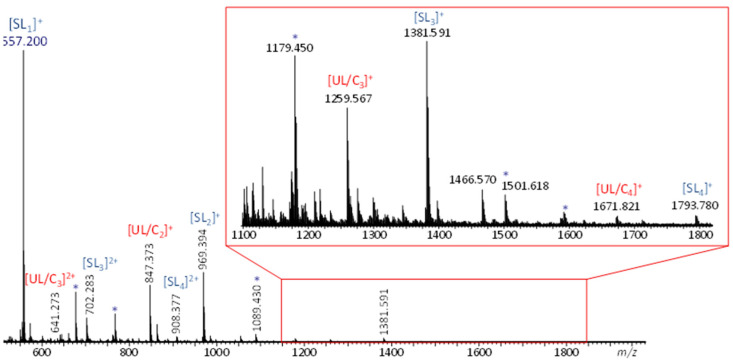
ESI-MS spectrum of dithiol-yne comb polymer. Peaks corresponding to singly and doubly charged unsaturated and saturated oligomers are marked accordingly (Na^+^ charges); byproducts formed because of the excess dithiol are marked with an asterisk.

Initial ESI-MS analysis of a dithiol-yne comb mixture prepared using a 2:1 molar equivalence of BDT to MeO-PEG_5_-Propargyl gave rise to two singly charged polymeric distributions (cf. Figure 2). One distribution corresponds to sodiated low-molecular-weight oligomers with the composition (C_18_H_36_O_6_S_2_)*_n_* and no nominal end groups, referred to as UL/C*_n_*. The other distribution, 122 Da higher, corresponds to the sodiated secondary product formed by chain termination with excess butanedithiol, referred to as SL*_n_*, which has a thiol end group at both extremities. Additionally, doubly charged oligomers of each architecture were also observed. Since the unsaturated linear product and the cyclized product are isomers with the same degree of unsaturation and the same exact mass, their architecture cannot be distinguished by ESI-MS alone. As a result, ESI-MS/MS and ESI-IM-MS experiments were carried out to gain more insight into the unknown oligomer architecture. In these experiments, the saturated linear species served as a pseudo-reference since these species must have a linear main chain architecture. As such, any differences or similarities between the ESI-MS/MS and ESI-IM-MS characteristics of the known saturated vs. the unknown unsaturated/cyclic oligomers can be used to decipher the oligomeric architecture. 

### 3.2. ESI-MS/MS Analysis

**Figure 3:**
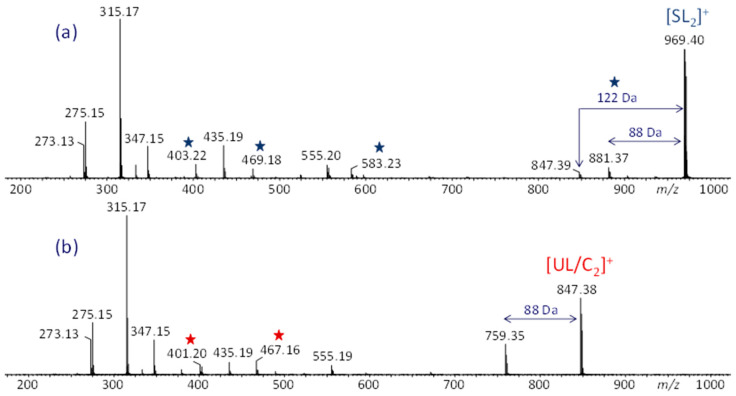
ESI-MS/MS spectra of the [M + Na]^+^ ions from the (**a**) saturated linear (SL) dimer and (**b**) unsaturated linear/cyclic (UL/C) dimer. Fragments characteristic for each structure are marked with blue or red asterisks, respectively. Adapted from ref. [13].

The sodiated dimers were selected first for MS/MS experiments as they were observed in the highest intensity in the ESI-MS spectrum (cf. Figure 2). The fragmentation patterns of both the UL/C dimer and SL dimer are similar (cf. Figure 3), with the most abundant peaks corresponding to product ions that can arise from either dimer by charge-induced or homolytic charge remote dissociation pathways at the PEG_5_ side chain [21]; the structures of these common fragments, observed at *m*/*z* 273, 275, 315, 347, 435, and 555, are summarized in Figure S2. Additionally, the loss of dioxane (C_4_H_8_O_2_, 88 Da) from the precursor ion is observed with both dimers, consistent with previously established fragmentation mechanisms of di-alkyl substituted PEG chains [21].

Unique and structurally diagnostic differences are evident when comparing the ESI-MS/MS results for the UC/L to the SL dimer. The [SL_2_]^+^ ion undergoes the loss of a BDT (122 Da) molecule likely from the extra pendant and forms a fragment of *m*/*z* 583 containing this pendant (cf. Scheme S1), both of which are not observed from [UL/C_2_]^+^, cf. Figure 3. The [SL_2_]^+^ dimer also dissociates to form a pair of complementary product ions, *m*/*z* 403 and 469, comprising opposite parts of the selected precursor ion (Scheme S1). Although the [UL/C_2_]^+^ dimer also forms an *m*/*z* 403 fragment, its relative intensity is lower than for [SL_2_]^+^; more importantly, [UL/C_2_]^+^ coproduces a different pair of complementary product ions, *m*/*z* 401 and 467, which are absent or near noise level in the MS/MS spectrum of [SL_2_]^+^ (cf. Figure 3). The unique MS/MS products of [UL/C_2_]^+^ are most readily rationalized from a cyclic architecture, as illustrated in Scheme S2 (vide infra). It is noteworthy that the complementary fragment pair observed from [UL/C_2_]^+^, viz. *m*/*z* 401 and 467, exhibits a higher degree of unsaturation than that from [SL_2_]^+^, viz. *m*/*z* 403 and 469, in keeping with the unsaturated nature of the UL/C precursor ion. 

The differences between the MS/MS results for the saturated vs. the unsaturated dimer provide information about the likely architecture of the unsaturated species. The unsaturated linear (UL) and saturated linear (SL) chains share common structural features: both contain a thiol end group that can be eliminated in the form of a BDT molecule, and both are capable of forming a fragment of *m*/*z* 469 via the same mechanism, as demonstrated in Schemes S1 and S3 for the UL and SL chain, respectively. However, several of the MS/MS fragments observed from [SL_2_]^+^ that would also be possible from [UL_2_]^+^, cf. Scheme S3, are not observed in the MS/MS spectrum of [UL/C]_2_^+^, suggesting that the unsaturated chain had cyclized (vide supra). 

Similar results can be observed from the ESI-MS/MS analysis of the sodiated UL/C and SL trimers (Figure S3). The most intense fragment for both species is the propylene-terminated PEG_5_ at *m*/*z* 315. Consistent with the dimer results, the loss of 122 Da from the SL trimer tail and an *m*/*z* 583 fragment encompassing this tail are once again observed. The fragments at *m*/*z* 881 from [SL_3_]^+^ and 879 from [UL/C_3_]^+^ are attributed to the same charge remote homolytic C-S bond cleavages yielding *m*/*z* 469 from [SL_2_]^+^ and 467 from [UL/C_2_]^+^, respectively, with the mass increase equivalent to the addition of one monomer unit (412 Da); for clarity, these dissociations are presented in Schemes S4 and S5, respectively (left side). Lastly, the SL trimer can lose one monomer unit to form the fragment at *m*/*z* 969, as illustrated in Scheme S4 (right side), whereas the UL/C species can undergo C-S homolytic cleavages at two nearby C-S bonds followed by recyclization to form a disulfide ring at *m*/*z* 967, as depicted in Scheme S5 (right side).

The unique fragments observed from [UL/C_3_]^+^ are best rationalized from the cyclic architecture [C_3_]^+^, as shown in Scheme S5. The isomeric [UL_3_]^+^ architecture should be able to lose a 122-Da molecule and form a fragment at *m*/*z* 881 because it contains the structural features required for these dissociations (cf. Scheme S6); the lack of these fragments in the corresponding MS/MS spectrum (Figure S3b) reinforces the conclusion that the trimer without an additional BDT monomer has cyclized. The collective differences in the MS/MS fragmentation patterns between the SL and UL/C dimers and trimers provide credible evidence that chains with a 1:1 molar ratio of BDT and PEG_5_ macromonomer have undergone intramolecular cyclization at low degrees of polymerization. 

### 3.3. ESI-IM-MS Analysis

**Figure 4:**
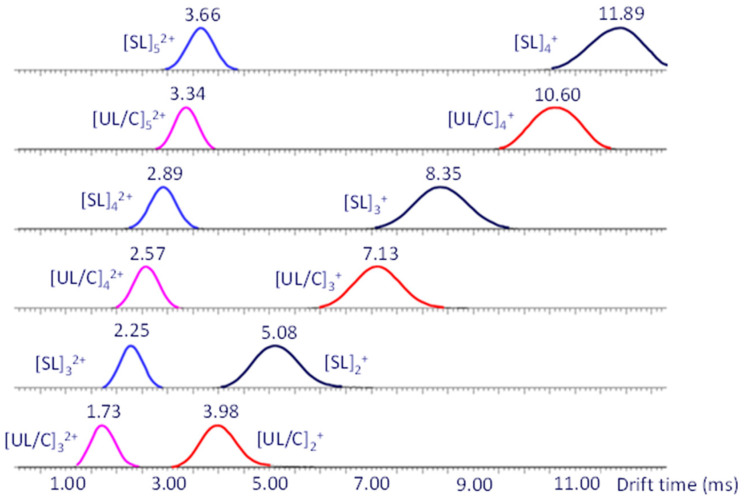
IM-MS mobilograms (drift time distributions) of singly and doubly charged dithiol-yne oligomers (Na^+^ charges) extracted from the 2D ESI-IM-MS plot (drift time vs. *m*/*z*). Singly sodiated dimers, trimers, and tetramers are observed at drift times between 3.98 and 11.89 ms; doubly sodiated trimers, tetramers, and pentamers are observed at drift times between 1.73 and 3.66 ms. Adapted from ref. [13].

Further characterization of the dithiol-yne oligomers was carried out using ESI-IM-MS. Orthogonal separation based on charge and shape that occurs within the IM cell allows for the distinction of ions with similar masses but different architectures. As shown in Figure 4, each dithiol-yne oligomer travels with a unique drift time through the IM cell. For each oligomer size, the UL/C species has a smaller drift time than the SL species, as expected if cyclization of the UL chain occurred. However, the shorter drift time of the UL oligomers could also be the result of their lower mass.

**Figure 5:**
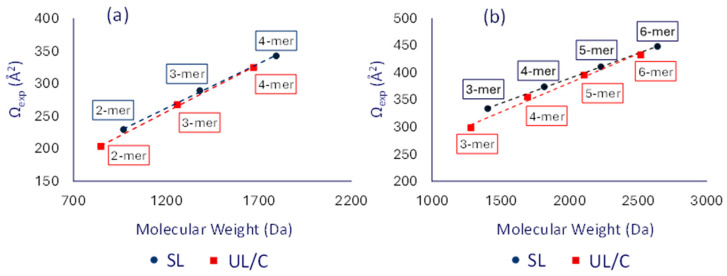
Plots of the average experimental ^TW^Ω_N2_→_He_ values for (**a**) the singly and (**b**) doubly charged dithiol-yne comb oligomers (Na^+^ charges). Blue color is used for the [SL*_n_*] and red color for the [UL/C*_n_*] chains.

To ensure that the observed differences in drift time were not solely a result of mass variations, plots of the measured collision cross-sections (Ω_exp_ values in Table S2) vs. the corresponding molecular weights were constructed for both singly as well as doubly charged [SL*_n_*] and [UL/C*_n_*] oligomers cf. Figure 5. When looking at the trend lines as opposed to individual Ω_exp_ values, the variation in oligomer mass is accounted for so that a direct architectural comparison can be made.

The trend lines for the saturated SL oligomers in Figure 5, shown in blue, lie consistently higher than those of the unknown UL/C oligomers, which are shown in red. For both singly as well as doubly charged ions, the distance between the two trend lines decreases with increasing oligomer size. Two explanations are possible for this observation. The first is that the small oligomers in the unsaturated product, such as dimers and trimers, undergo facile intramolecular cyclization, but the larger oligomers do not, thus causing the Ω trend line of the UL/C chains to approach that of the SL chains. The second explanation is that chain flexibility starts to play a significant role in the observed collision cross-section as oligomer size increases. Specifically, longer linear chains would be able to form more compact structures than isomeric cyclic chains, which are spatially constricted and more rigid. To further investigate these experimental trends, theoretical Ω data for the unsaturated linear (UL), cyclic (C), and saturated linear (SL) architecture were obtained using molecular modeling, as discussed in the following section.

### 3.4. Molecular Modeling 

Average theoretical Ω values (in He) were calculated from 50 candidate structures for each oligomer architecture (Table S2). A comparison of the Ω_exp_ and Ω_theo_ data in Table S2 clearly shows that the theory overestimates the collision cross-sections of Na^+^-cationized dithiol-yne comb copolymers by, on average, ±22%. This result is attributed to the molecular dynamics simulations used, which often lead to larger conformations and smaller compactness than IM-MS experiments [44,45]. More rigorous computational methods, like density functional theory or molecular orbital calculations, can provide better results, but even in these cases, scaling of the Ω_theo_ data is necessary for satisfactory agreement with the experiment if the system under study contains third-row or heavier elements, as is the case with our dithiol-yne comb polymers which contain multiple sulfurs and a sodium cation [46,47]. Moreover, the force field used for MD calculations is not specifically designed for the polymer molecules currently under investigation, which may lead to discrepancies when compared to experimental results. This is especially true when focusing on single-molecule characterization rather than bulk properties, resulting in less accuracy. Although DFT results would be ideal, they are computationally intractable for some of these cases. Nevertheless, MD results can provide useful trends within a more reasonable computational time. For the ions with multiple sulfur atoms investigated here, a scaling factor of 80% affords good agreement between theoretical predictions and experimental results (error < ±4%), cf. Table S2. However, because of this uncertainty and the very similar collision cross-sections of the [C*_n_*] and [UL*_n_*] chains, architectural differentiation will be discussed solely in terms of Ω_theo_ trends and not of the corresponding absolute collision cross-sections.

**Figure 6:**
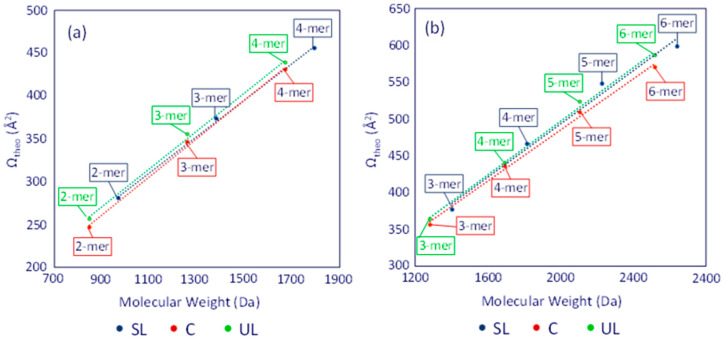
Plots of the average theoretical Ω_He_ values for (**a**) the singly and (**b**) doubly charged dithiol-yne comb oligomers (Na^+^ charges). Blue color is used for the [SL*_n_*], red color for the [C*_n_*], and green color for the [UL*_n_*] chains. Adapted from ref. [13].

The average Ω_theo_ values of the dithiol-yne oligomers were plotted as a function of molecular weight, similar to the experimental results, cf. Figure 6. When inspecting the theoretical Ω values, two distinct trends emerge. First, the theoretical trend line for the unsaturated linear (UL) structures lies above the saturated linear (SL) trend line. This result reveals that the terminal double bond in the UL architecture (cf. Scheme 1) adds conformational rigidity that limits the folding possible in [UL*_n_*]^+^ or [UL*_n_*]^2+^ chains compared to [SL*_n_*]^+^ or [SL*_n_*]^2+^ chains, respectively. Secondly, the theoretical trend lines for both the singly and doubly charged cyclic (C) structures lie below the corresponding SL trend lines, indicating that the macrocyclic structure (Scheme 1) can attain a higher degree of compactness than the other two architectures.

The consistently higher position (in the y-axis) of the trend line for the unsaturated linear (UL) architecture is opposite to the experimentally observed Ω values of the unknown oligomers (cf. Figure 5 vs. Figure 6), corroborating that the unsaturated chains examined in this study (up to the hexamer) have cyclized. The theoretical predictions in Figure 6 also help clarify the trends observed in the IM-MS experiments. At smaller oligomer sizes, the saturated linear (SL) chain can expand more easily than the cyclic (C) chain, resulting in higher Ω values. As oligomer size increases, the SL chain can coil more easily, resulting in more compact globule structures than possible with the cyclic chain (Figure S4 and Tables S3–S6). This justifies the convergence in the Ω_theo_ trend observed at the size of singly charged tetramers and doubly charged hexamers.

## 4. Conclusions

In this study, dithiol-yne comb polymers were synthesized by a recently developed branching-controlled technique utilizing an alkyne macromonomer and a dithiol linker [9]. With an excess of the dithiol linker, two distinct products were identified by ESI-MS analysis: one distribution of unsaturated oligomers and the other of fully saturated linear oligomers. ESI-MS/MS analysis of both products resulted in six common fragments formed mainly by bond cleavages at one of the PEG_5_ tails. In addition to these similar fragmentation pathways, several distinct fragments were observed for the saturated linear oligomers, which were not observed in the unsaturated oligomers. These results and the proposed fragmentation mechanisms suggest that the backbone of the unsaturated product has undergone intramolecular cyclization. 

IM-MS experiments provided complementary and comparable information to that deduced from the ESI-MS/MS experiments. The Ω values of the singly and doubly charged unsaturated linear/cyclic comb oligomers indicated chain cyclization for the degrees of polymerization examined in this work (dimer to hexamer). The theoretical predictions obtained from molecular dynamics simulated structures using the trajectory method in MOBCAL (Ω_theo_) underestimated the compactness of dithiol-yne chains (by ~22%). This discrepancy is likely due to the presence of several third-row atoms in these species and the lack of accurately trained force field parameters for the current molecules; however, scaling of the Ω_theo_ values by 80% yielded good agreement with the corresponding Ω_exp_ data (<±4% average error).

Although our combined MS/MS and IM-MS investigation provided strong evidence that smaller unsaturated dithiol-yne oligomers with a 1:1 molar ratio of BDT and PEG_5_ have cyclized, the fate of longer chains with larger degrees of polymerization (>10 monomer units) remains unknown. Future work should be aimed at carrying out similar studies on higher molecular weight oligomers isolated via GPC fraction collection techniques.

## Data Availability

Data will be made available on request.

## Supplementary Materials

The following supporting information can be downloaded at https://www.mdpi.com/article/10.3390/polym16121665/s1: IM-MS calibration data and curves (Figure S1 and Table S1); common MS/MS fragment structures (Figure S2); trimer MS/MS spectra (Figure S3); simulated dimer and tetramer structures and their XYZ coordinates (Figure S4 and Tables S3–S6); MS/MS fragmentation pathways (Schemes S1–S6); collision cross-section data (Table S2).
