# Supplementary material for: Elucidation of Dithiol-yne Comb Polymer Architectures by Tandem Mass Spectrometry and Ion Mobility Techniques
Source: Polymers (Basel). 2024 Jun 12;16(12):1665. doi: 10.3390/polym16121665 (PMC11207239; doi:10.3390/polym16121665)
Supplement: Supplementary file 1 [file polymers-16-01665-s001.zip › polymers-2887676-supplementary.pdf]

Supporting Information

Elucidation of Dithiol-yne Comb Polymer Architectures by Tandem Mass Spectrometry and Ion Mobility Techniques

Kayla Williams-Pavlantos [1], Abdol Hadi Mokarizadeh [2], Brennan J. Curole [3], Scott M. Grayson [3,*], Mesfin Tsige [2] and Chrys Wesdemiotis [1,2,*]

1 Department of Chemistry, University of Akron, Akron, OH 44325, USA; knw55@uakron.edu
2 School of Polymer Science and Polymer Engineering, University of Akron, Akron, OH 44325, USA; am555@uakron.edu (A.H.M.); mtsige@uakron.edu (M.T.)
3 Department of Chemistry, Tulane University, New Orleans, LA 70118, USA; bcurole1@tulane.edu
* Correspondence: sgrayson@tulane.edu (S.M.G.); wesdemiotis@uakron.edu (C.W.); Tel.: +1-504-862-8135 (S.M.G.); +1-330-972-7699 (C.W.)

Table of contents:

Figure S1. IM-MS calibration curves.

Figure S2. Common fragments in the ESI-MS/MS spectra of sodiated saturated linear (SL) and unsaturated linear/cyclic (UL/C) dithiol-yne comb oligomers.

Figure S3. ESI-MS/MS spectra of the sodiated saturated linear (SL) and unsaturated linear/cyclic (UL/C) trimers.

Figure S4. Simulated structures and collision cross-sections of the sodiated cyclic (C) and saturated linear (SL) dithiol-yne dimer and tetramer.

Scheme S1. Fragmentation pathways of the sodiated saturated linear (SL) dimer.

Scheme S2. Fragmentation pathways of the sodiated cyclic (C) dimer.

Scheme S3. Fragmentation pathways expected from the sodiated unsaturated linear (UL) dimer.

Scheme S4. Fragmentation pathways of the sodiated saturated linear (SL) trimer.

Scheme S5. Fragmentation pathways of the sodiated cyclic (C) trimer.

Scheme S6. Fragmentation pathways expected from the sodiated unsaturated linear (UL) trimer.

Table S1. Corrected drift time and collision cross-section data used for IM-MS calibration.

Table S2. Experimental and theoretical collision cross-sections.

Table S3-S6. XYZ coordinates for the $[C_n]^+$ and $[SL_n]^+$ structures in Figure S4.

References

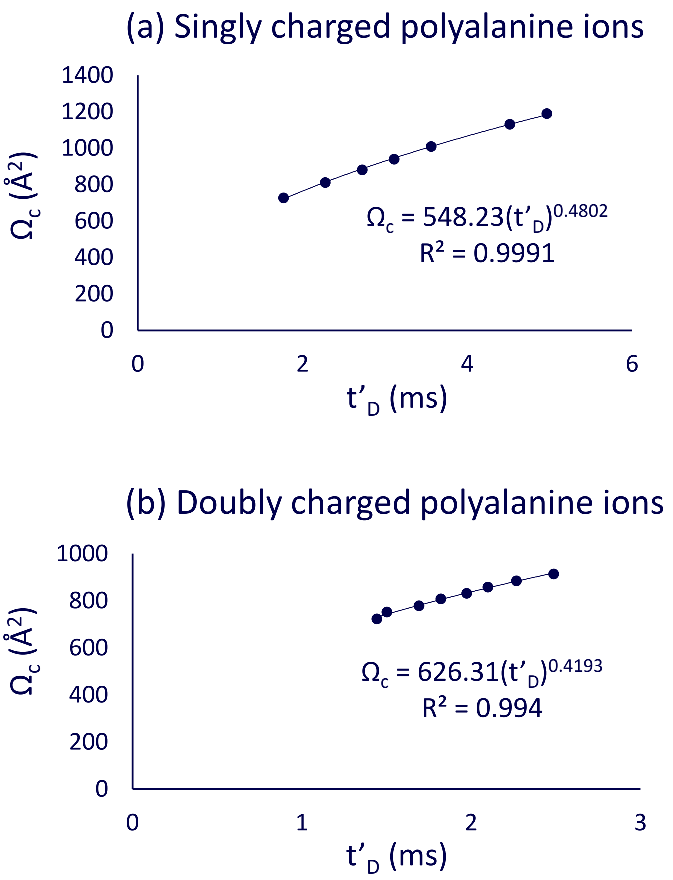

Figure S1. Singly and doubly charged IM-MS calibration curves constructed from the corrected drift time (t'_D) and corrected collision cross-section (Ω_c) data presented in Table S1.

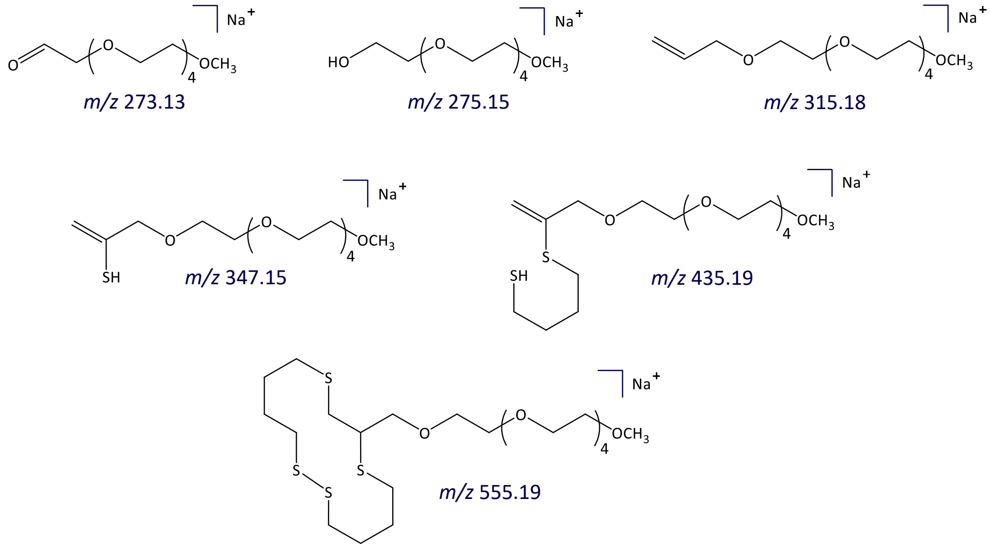

Figure S2. Common fragments in the ESI-MS/MS spectra of sodiated $[SL_2]^+$ (*m/z* 969.40) and $[UL/C_2]^+$ (*m/z* 847.38), formed by dissociation pathways initiated by charge-induced C-O ether bond cleavage and/or charge-remote homolytic C-S bond cleavage [1].

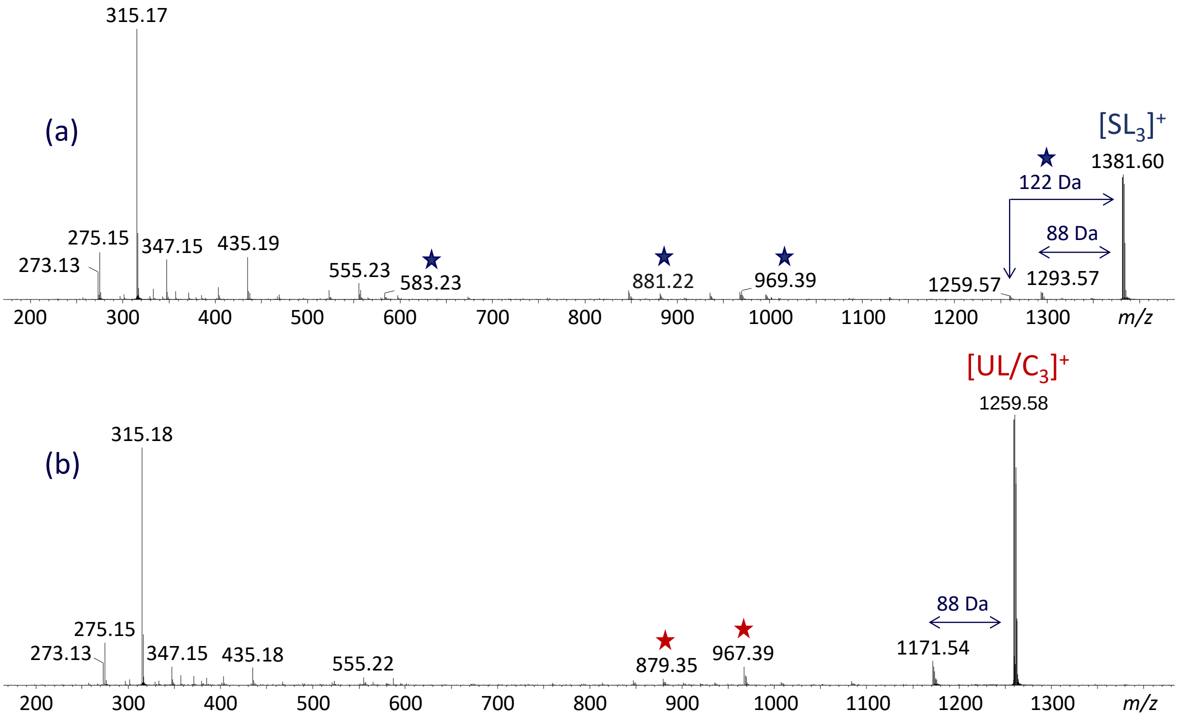

Figure S3. ESI-MS/MS spectra of the $[M + Na]^+$ ions from the (a) saturated linear (SL) trimer and (b) unsaturated linear/cyclic (UL/C) trimer. Fragments characteristic for each structure are marked with blue or red asterisks, respectively. Adapted from ref. [2]

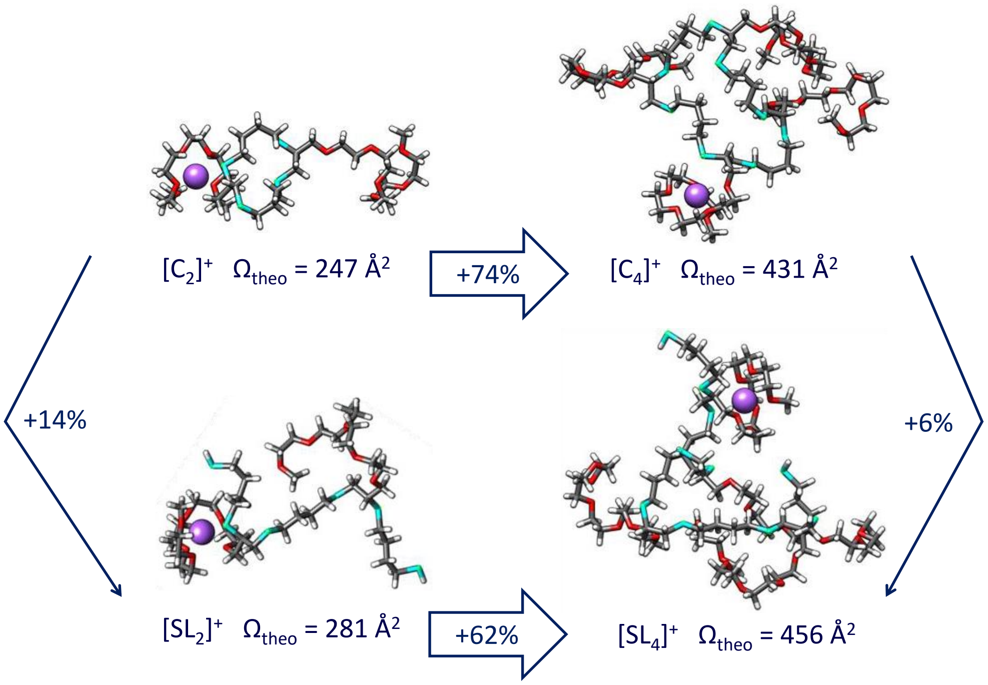

Figure S4. Simulated structures and collision cross-sections of singly sodiated cyclic (C) dimers and tetramers (top) and saturated linear (SL) dimers and tetramers (bottom). The Na^+ ion, shown in purple, is attached to one PEG_5 pendant. The CCS increase (in %) from dimer to tetramer is smaller for the linear chain, which is more flexible and can more easily form compact globule structures. Similarly, the CCS increase from cyclic (C) to saturated linear (SL) oligomer of the same size is smaller for the longer chain, which can more easily coil to maximize intermolecular stabilizing interactions. Adapted from ref. [2].

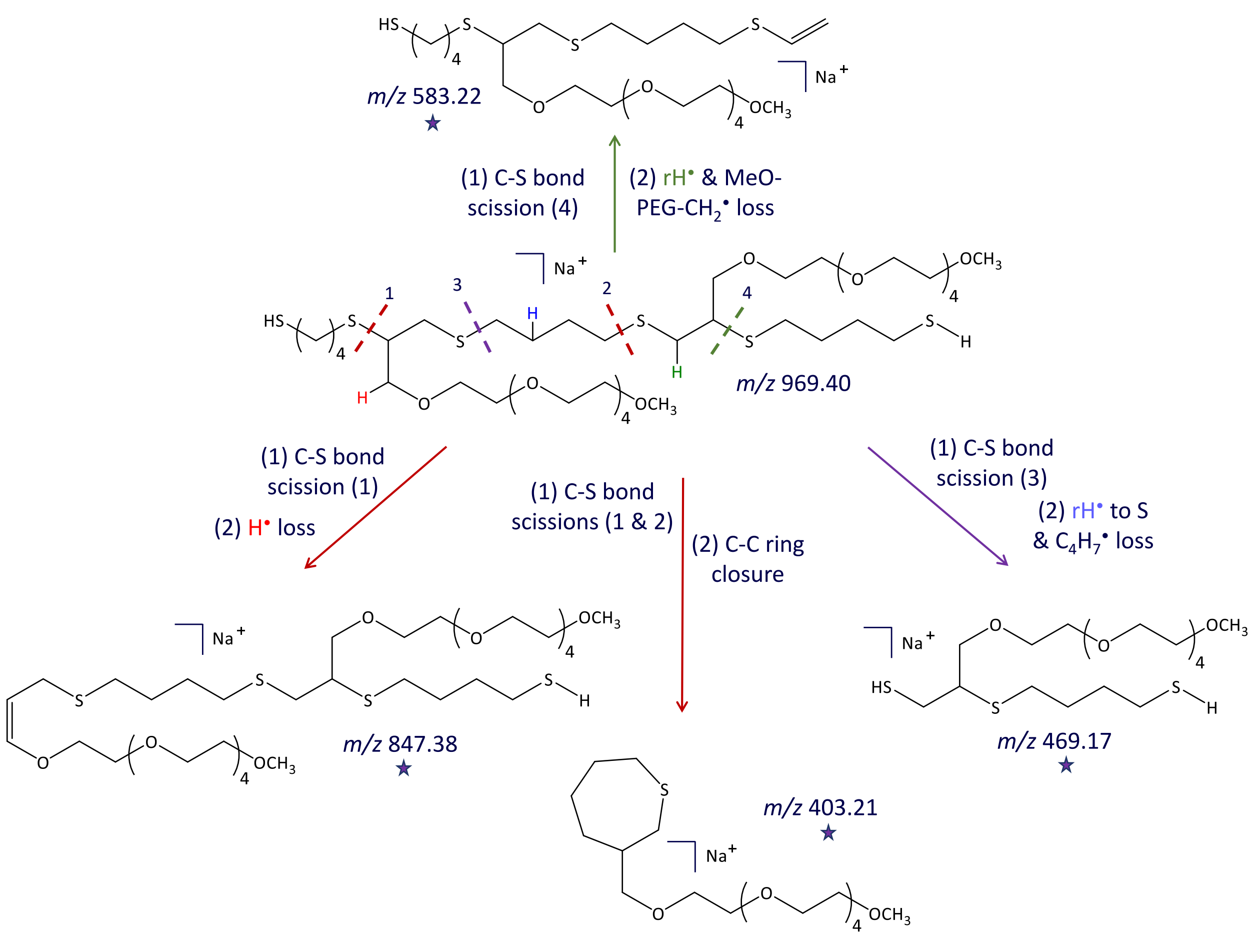

Scheme S1. Proposed fragmentation pathways of the sodiated saturated linear dimer, $[SL_2]^+$, leading to fragment ions indictive of the linear architecture. All are initiated by homolytic C-S bond cleavages and are accompanied by H• loss, H• transfer (rearrangement), or radical recombination (ring closure). (All Schemes include calculated *m*/*z* values, whereas the Figures include measured *m*/*z* values.)

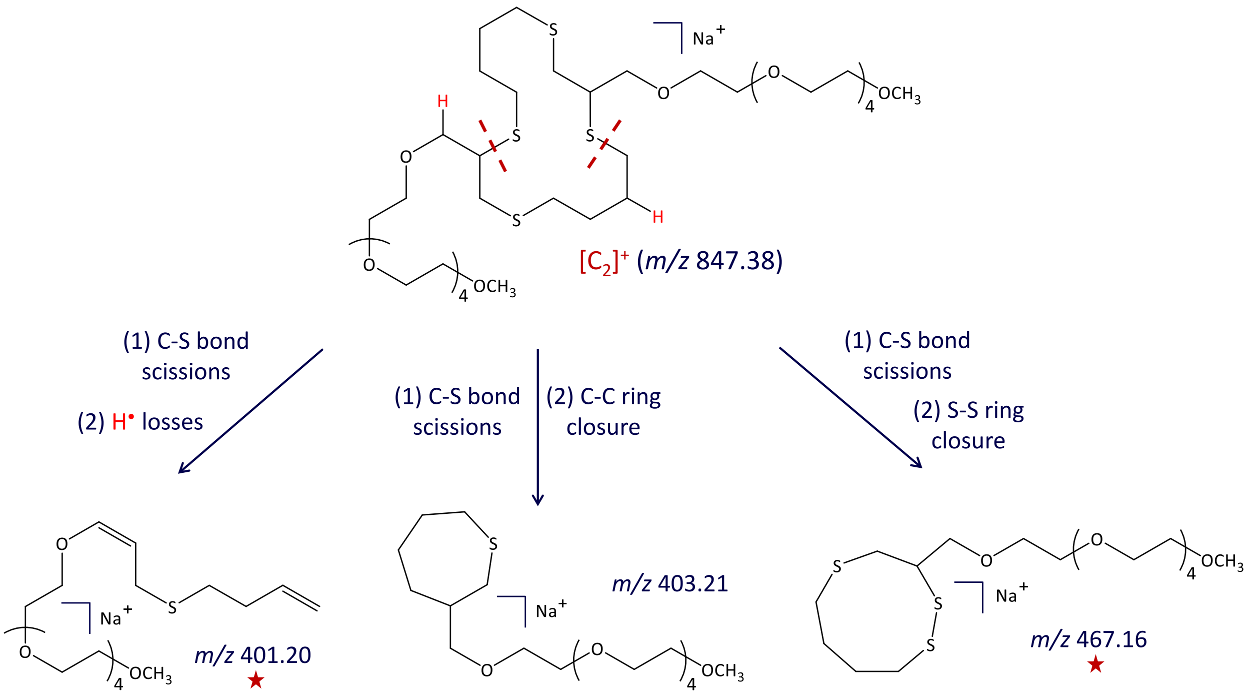

Scheme S2. Proposed fragmentation pathways of the sodiated cyclic dimer, $[C_2]^+$, leading to fragment ions indictive of the cyclic architecture. All are initiated by homolytic C-S bond cleavages and are accompanied by H• losses or radical recombination (ring closure).

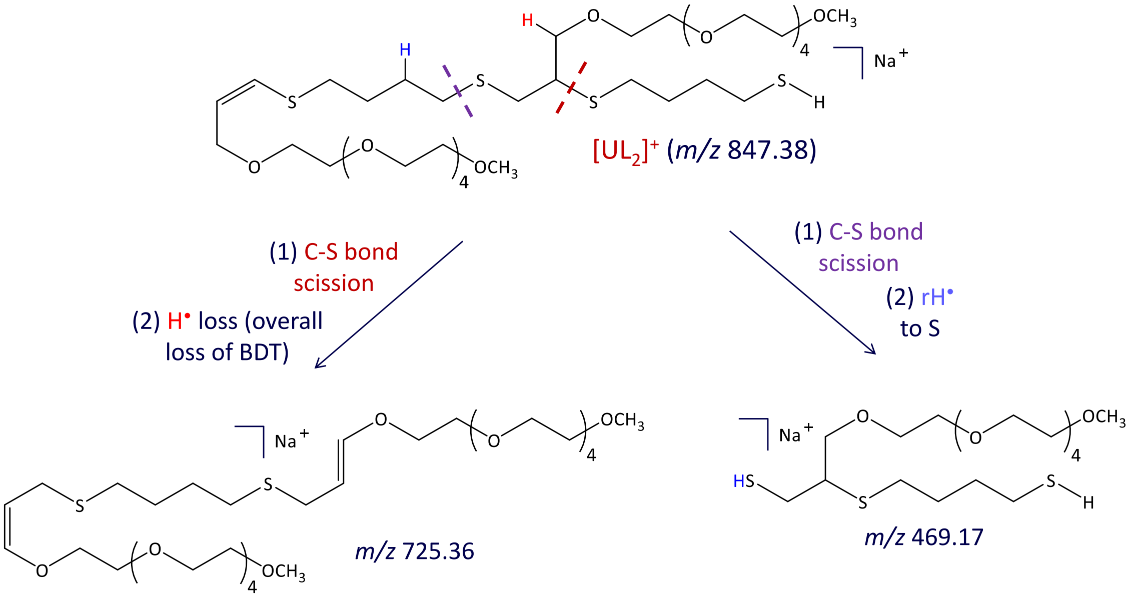

Scheme S3. Fragmentation pathways expected from the sodiated unsaturated linear dimer, $[UL_2]^+$, based on the MS/MS fragmentation pattern observed from $[SL_2]^+$. These fragments are not observed in the

MS/MS spectrum of Figure 3b, strongly suggesting that the dimer analyzed was cyclized and had the $[C_2]^+$ connectivity.

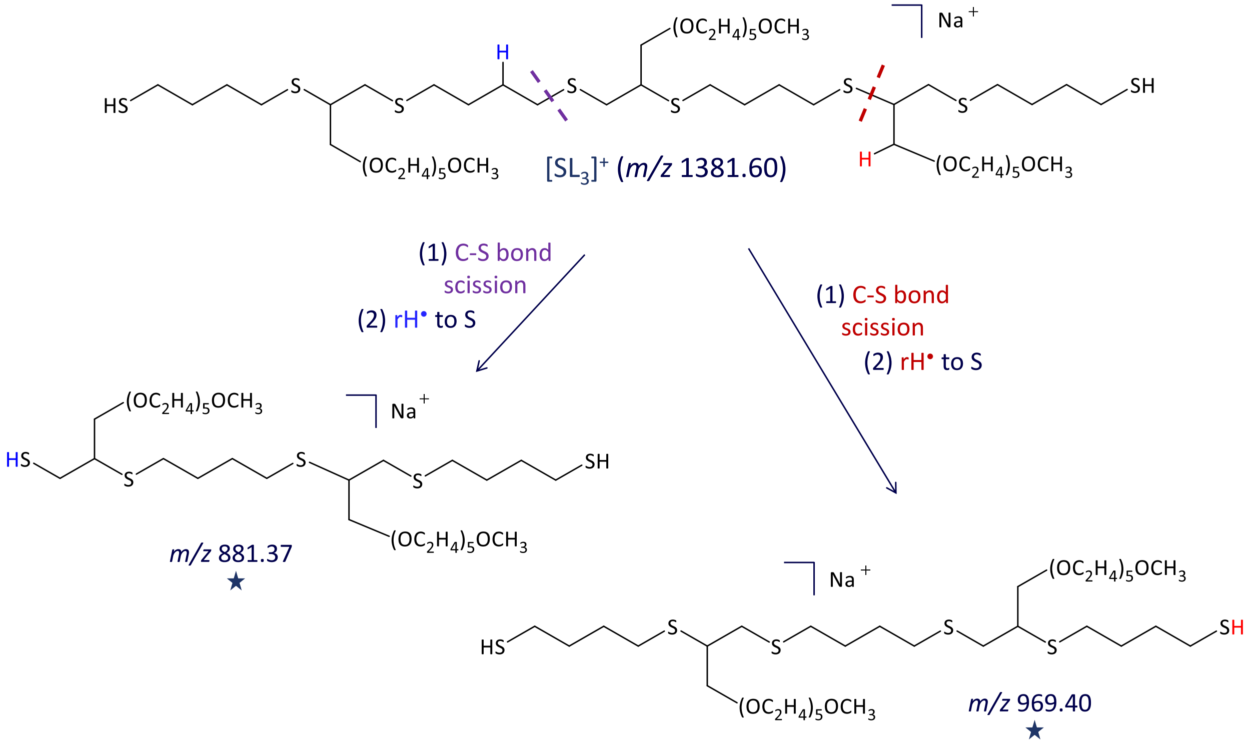

Scheme S4. Proposed fragmentation pathways of the sodiated saturated linear trimer, $[SL_3]^+$, leading to fragment ions indictive of the linear architecture. They are initiated by homolytic C-S bond cleavages and are accompanied by H• transfer (rearrangement) to the emerging S• radical from the detached neutral loss.

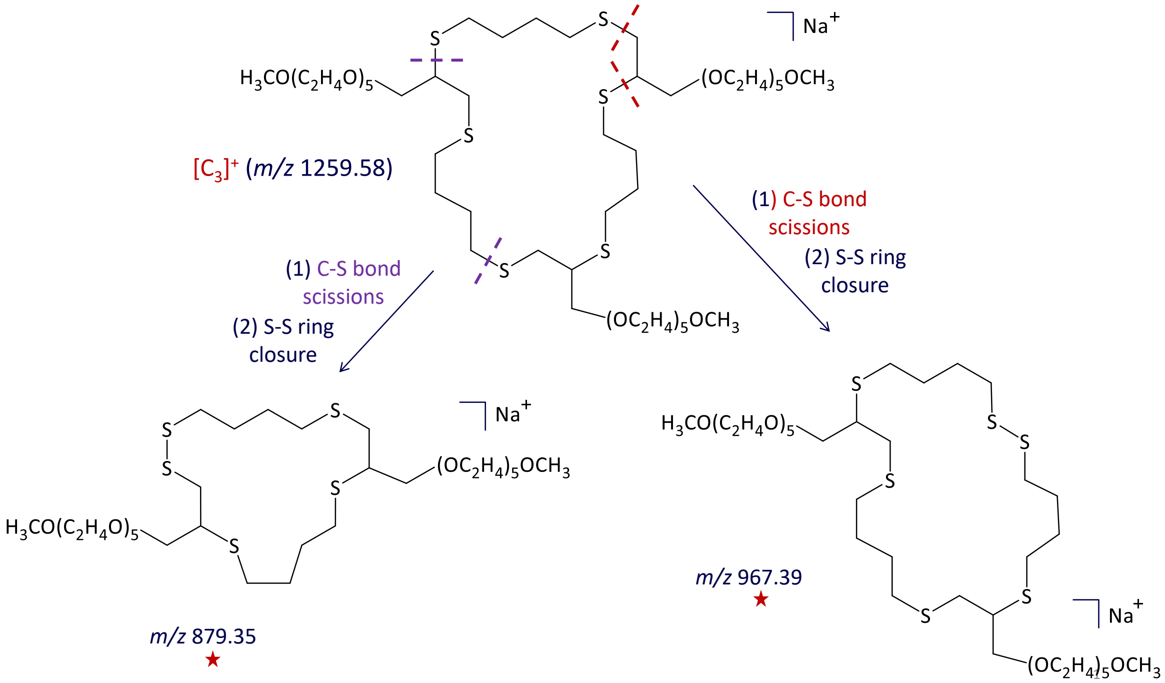

Scheme S5. Proposed fragmentation pathways of the cyclic trimer, $[C_3]^+$, leading to fragment ions indictive of the cyclic architecture. They are initiated by homolytic C-S bond cleavages followed by radical recombination (ring closure).

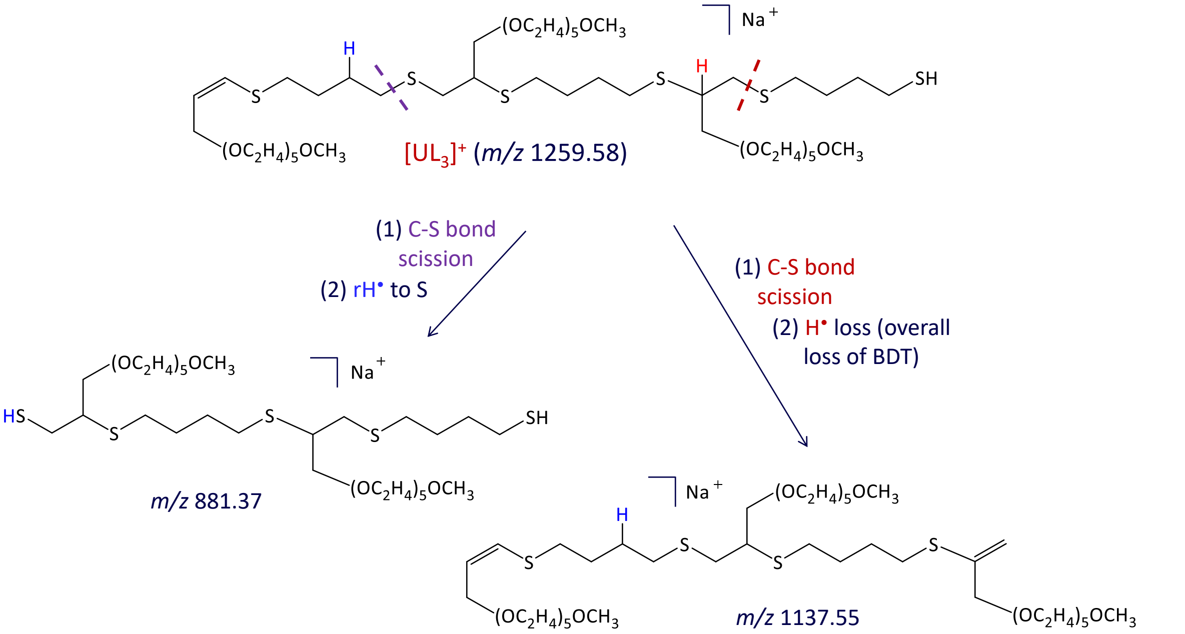

Scheme S6. Fragmentation pathways expected from the sodiated unsaturated linear trimer, $[UL_3]^+$, based on the MS/MS fragmentation pattern observed from $[SL_3]^+$. These fragments are not observed in the

MS/MS spectrum of Figure S3b, strongly suggesting that the trimer analyzed was cyclized and had the $[C_3]^+$ connectivity.

Table S1. Corrected drift times and corrected collision cross-sections for the polyalanine $[(Ala)_n + zH]^{z+}$ oligomers used for the calibration curves in Figure S1.

z	MW	*m/z*	t'$_D$ (ms) [a]	Ω (Å^2) [b]	Ω$_c$ (Å^2) [c]
1	515.27	516.28	1.768	141	726.74
1	586.31	587.32	2.276	157	811.75
1	657.35	658.35	2.724	170	881.14
1	728.38	729.39	3.112	181	940.023
1	799.42	800.43	3.560	194	1009.201
1	941.49	942.50	4.517	217	1131.75
1	1012.53	1013.24	4.965	228	1190.33
2	1296.43	649.22	1.444	276	722.59
2	1367.45	684.74	1.503	287	751.81
2	1438.76	720.39	1.692	297	778.39
2	1509.51	755.77	1.821	308	807.58
2	1580.83	791.42	1.974	317	831.52
2	1651.86	826.94	2.099	327	858.07
2	1722.90	862.46	2.269	337	884.62
2	1793.61	897.82	2.488	348	913.78
2	1864.98	933.50	2.634	358	940.31

[a] Corrected drift time, $t'_D = t_D - 1.4(m/z)^{0.5}/1000$, where t_D is the measured drift time in ms.

[b] Published $^{DT}\Omega_{He}$ values, acquired using drift-tube (DT) IM-MS and He as buffer gas [3].

[c] Corrected Ω, Ω_c = $^{DT}\Omega_{He}$ ($\mu^{0.5}/z$), where μ is the reduced mass of the polyalanine ion and the buffer gas used in the TWIM cell (N_2).

Table S2. Average experimental and theoretical collision cross-section (Ω) values with relative errors.

Oligomer ion [a]	Architecture (*m/z*)	Ω_{exp} (Å²) [b]	Ω_{theo} (Å²) [c]	Ω_{theo} (Å²) [d] scaled 80%	% Δ (±) [e]	% Δ (±) [f] scaled 80%
[2-mer]$^+$	C (847.4)	203 (±0.0)	247 (±16)	198	-18	-3
	UL (847.4)		257 (±13)	206	-21	1
	SL (969.4)	229 (±0.6)	281 (±12)	225	-19	-2
[3-mer]$^+$	C (1259.6)	267 (±0.5)	346 (±18)	277	-23	4
	UL (1259.6)		356 (±28)	285	-25	6
	SL (1381.6)	289 (±0.0)	374 (±18)	299	-23	3
[4-mer]$^+$	C (1671.8)	324 (±0.5)	431 (±20)	345	-25	6
	UL (1671.8)		439 (±18)	351	-26	8
	SL (1793.8)	342 (±1.5)	456 (±20)	365	-25	6
[3-mer]$^{2+}$	C (641.3)	299 (±2.1)	356 (±10)	285	-16	-5
	UL (641.3)		364 (±11)	291	-18	-3
	SL (702.3)	334 (±2.1)	377 (±12)	302	-11	-11
[4-mer]$^{2+}$	C (847.4)	355 (±0.0)	436 (±19)	349	-19	-2
	UL (847.4)		440 (±17)	352	-19	-1
	SL (908.4)	374 (±1.8)	466 (±18)	373	-20	0
[5-mer]$^{2+}$	C (1053.5)	395 (±0.0)	509 (±20)	407	-22	3
	UL (1053.5)		523 (±21)	418	-24	6
	SL (1114.5)	411 (±0.0)	548 (±26)	438	-25	6
[6-mer]$^{2+}$	C (1259.6)	433 (±1.7)	570 (±26)	456	-24	5
	UL (1259.6)		582 (±24)	466	-26	7
	SL (1320.6)	448 (±1.2)	598 (±25)	478	-25	6

[a] Singly or doubly sodiated ions.

[b] Average experimental $^{TW}\Omega_{N2\rightarrow He}$ value of UL/C or SL oligomer from triplicate measurements (std. deviation in parenthesis).

[c] Average calculated Ω_{He} value of 50 geometry optimized candidate structures with C, UL, or SL architecture (± one std. deviation).

[d] Scaled collision cross-sections; such scaling is often necessary with larger ions containing 3rd-row or heavier elements to obtain satisfactory agreement between measured and theoretically predicted collision cross-sections [4,5].

[e] Ω_{theo} – Ω_{exp} in %. Average difference ±22% for all data.

[e] Ω_{theo} – Ω_{exp} in %. Average difference ±<4% for all data.

Table S3. XYZ coordinates for structure $[C_2]^+$ shown in Figure S4.

C	-1289.32	-729.22	-60.2
O	-1288.74	-730.49	-60.44
C	-1287.47	-730.42	-61.05
C	-1286.9	-731.8	-61.24
O	-1285.57	-731.66	-61.72
C	-1284.86	-732.88	-61.93
C	-1284.35	-733.55	-60.68
O	-1285.38	-734.07	-59.83
C	-1285.56	-735.49	-59.87
C	-1286.1	-736.05	-61.15
O	-1287.35	-735.44	-61.45
C	-1287.99	-735.99	-62.6
C	-1287.41	-735.59	-63.93
O	-1286.23	-736.34	-64.18
C	-1285.64	-736.13	-65.46
C	-1284.73	-734.94	-65.56
O	-1285.48	-733.75	-65.38
C	-1284.71	-732.59	-65.62
H	-1289.43	-728.65	-61.13
H	-1288.69	-728.63	-59.51
H	-1286.75	-729.84	-60.43
H	-1287.51	-729.92	-62.04
H	-1287.54	-732.38	-61.93
H	-1286.94	-732.32	-60.26
H	-1285.45	-733.6	-62.52
H	-1284	-732.59	-62.55
H	-1283.81	-732.8	-60.08
H	-1283.62	-734.32	-60.95
H	-1286.3	-735.7	-59.07
H	-1284.63	-736.01	-59.58
H	-1286.2	-737.14	-61.04
H	-1285.37	-735.88	-61.97
H	-1289.03	-735.64	-62.53
H	-1288.02	-737.09	-62.56
H	-1287.2	-734.5	-63.92
H	-1288.17	-735.77	-64.71
H	-1286.41	-736.06	-66.25
H	-1285.06	-737.04	-65.66
H	-1284.25	-734.95	-66.56
H	-1283.93	-735.02	-64.8
H	-1283.81	-732.55	-64.98
H	-1285.35	-731.73	-65.37
H	-1284.4	-732.51	-66.67
C	-1294.01	-728.74	-53.83
S	-1294.3	-727.18	-54.73
C	-1292.73	-727.02	-55.62
C	-1292.81	-726.03	-56.79
C	-1291.45	-725.79	-57.48
C	-1290.75	-727.06	-57.99
S	-1291.68	-727.9	-59.3
C	-1290.76	-729.47	-59.54
C	-1290.65	-730.32	-58.24
S	-1292.25	-730.61	-57.42
C	-1291.77	-731.5	-55.91
C	-1291.01	-730.63	-54.91
C	-1290.9	-731.27	-53.52
C	-1292.24	-731.41	-52.75
S	-1292.67	-730	-51.68
C	-1292.94	-728.56	-52.77
C	-1295.39	-729.28	-53.27
O	-1296.41	-729.34	-54.27
C	-1296.22	-730.4	-55.2
C	-1297.29	-730.26	-56.23
O	-1297.37	-728.94	-56.76
C	-1296.42	-728.65	-57.78
C	-1296.62	-727.22	-58.16
O	-1296.48	-726.47	-56.96
C	-1296.74	-725.08	-57.11
C	-1296.56	-724.45	-55.77
O	-1297.44	-725.1	-54.86
C	-1297.25	-724.71	-53.5
C	-1298.2	-725.52	-52.69
O	-1297.91	-726.89	-52.95
C	-1298.92	-727.8	-52.52
C	-1300.04	-727.93	-53.5
O	-1299.47	-728.11	-54.79
C	-1300.48	-728.25	-55.78
H	-1295.26	-730.26	-52.77
H	-1295.77	-728.59	-52.5
H	-1296.29	-731.39	-54.7
H	-1295.23	-730.35	-55.68
H	-1298.27	-730.44	-55.75
H	-1297.19	-731.01	-57.04
H	-1296.56	-729.32	-58.65
H	-1295.39	-728.79	-57.42
H	-1297.62	-727.09	-58.59
H	-1295.89	-726.89	-58.92
H	-1297.78	-724.92	-57.45
H	-1296.07	-724.62	-57.86
H	-1296.76	-723.37	-55.8
H	-1295.51	-724.57	-55.46
H	-1297.43	-723.62	-53.37
H	-1296.21	-724.91	-53.17
H	-1299.23	-725.26	-52.99
H	-1298.12	-725.3	-51.61
H	-1299.29	-727.55	-51.51
H	-1298.42	-728.78	-52.43
H	-1300.68	-727.02	-53.48
H	-1300.69	-728.78	-53.23
H	-1301.12	-727.35	-55.86
H	-1301.13	-729.12	-55.58
H	-1299.98	-728.41	-56.74
H	-1293.64	-729.47	-54.57
H	-1292.46	-728.01	-56.01
H	-1291.95	-726.71	-54.91
H	-1293.19	-725.06	-56.43
H	-1293.54	-726.4	-57.53
H	-1290.78	-725.28	-56.76
H	-1291.58	-725.09	-58.32
H	-1290.57	-727.76	-57.15
H	-1289.76	-726.79	-58.39
H	-1291.35	-730.05	-60.27
H	-1289.96	-729.83	-57.54
H	-1290.2	-731.3	-58.48
H	-1291.19	-732.4	-56.18
H	-1292.72	-731.86	-55.47
H	-1291.48	-729.64	-54.83
H	-1289.99	-730.46	-55.31
H	-1290.19	-730.71	-52.89
H	-1290.46	-732.28	-53.65
H	-1292.15	-732.25	-52.04
H	-1293.08	-731.65	-53.41
H	-1291.98	-728.27	-53.22
H	-1293.23	-727.75	-52.08
Na	-1297.37	-727.37	-55.06

Table S4. XYZ coordinates for structure $[C_4]^+$ shown in Figure S4.

C	40.737	-301.869	-370.652
O	40.671	-300.455	-370.532
C	41.467	-299.814	-371.524
C	41.459	-298.332	-371.327
O	42.011	-298.014	-370.049
C	42.509	-296.683	-369.934
C	41.477	-295.589	-370.006
O	40.951	-295.318	-371.309
C	41.716	-294.413	-372.102
C	40.925	-294.017	-373.319
O	40.918	-294.963	-374.393
C	39.945	-295.989	-374.228
C	40.122	-297.012	-375.322
O	39.346	-298.201	-375.136
C	37.976	-298.119	-375.554
C	37.741	-298.516	-376.982
O	38.193	-297.475	-377.831
C	38.076	-297.783	-379.205
H	41.108	-300.063	-372.54
H	42.517	-300.156	-371.462
H	40.428	-297.945	-371.403
H	42.037	-297.859	-372.14
H	43.309	-296.494	-370.671
H	42.979	-296.648	-368.937
H	41.879	-294.672	-369.539
H	40.608	-295.897	-369.405
H	42.678	-294.858	-372.413
H	41.949	-293.505	-371.518
H	41.349	-293.101	-373.76
H	39.899	-293.771	-372.992
H	38.923	-295.57	-374.246
H	40.065	-296.505	-373.261
H	41.17	-297.346	-375.306
H	39.944	-296.547	-376.307
H	37.558	-297.112	-375.389
H	37.438	-298.802	-374.879
H	36.664	-298.704	-377.14
H	38.272	-299.465	-377.178
H	38.432	-296.901	-379.756
H	37.032	-297.98	-379.506
H	38.698	-298.647	-379.497
C	32.723	-298.404	-359.124
O	31.927	-299.007	-358.112
C	30.86	-298.168	-357.666
C	31.238	-297.211	-356.562
O	30.141	-296.326	-356.403
C	30.214	-295.475	-355.263
C	31.268	-294.406	-355.286
O	32.577	-294.939	-355.063
C	33.49	-293.899	-354.688
C	33.981	-293.047	-355.824
O	34.839	-293.826	-356.658
C	34.907	-293.425	-358.031
C	33.911	-294.119	-358.913
O	32.58	-293.672	-358.669
C	31.702	-294.362	-359.547
C	30.276	-293.937	-359.328
O	29.473	-294.751	-360.167
C	28.094	-294.566	-359.923
H	30.068	-298.841	-357.3
H	30.419	-297.585	-358.493
H	32.151	-296.654	-356.838
H	31.463	-297.766	-355.634
H	30.32	-296.053	-354.326
H	29.224	-294.995	-355.226
H	31.009	-293.682	-354.493
H	31.226	-293.862	-356.245
H	34.36	-294.4	-354.239
H	33.057	-293.271	-353.889
H	34.546	-292.183	-355.433
H	33.122	-292.645	-356.385
H	35.924	-293.698	-358.355
H	34.813	-292.33	-358.145
H	33.994	-295.206	-358.73
H	34.184	-293.944	-359.97
H	31.76	-295.454	-359.391
H	31.965	-294.177	-360.605
H	30.008	-294.06	-358.263
H	30.156	-292.866	-359.574
H	27.79	-293.512	-360.035
H	27.549	-295.163	-360.668
H	27.809	-294.918	-358.916
C	38.033	-302.382	-362.503
O	38.536	-303.527	-361.812
C	38.015	-303.566	-360.485
C	38.255	-304.918	-359.908
O	37.514	-305.836	-360.704
C	37.504	-307.181	-360.226
C	38.8	-307.885	-360.486
O	39.207	-307.525	-361.803
C	40.492	-308.003	-362.203
C	41.099	-306.958	-363.093
O	40.162	-306.403	-364.019
C	39.983	-307.218	-365.176
C	39.007	-306.513	-366.048
O	37.815	-306.326	-365.291
C	37.007	-305.287	-365.839
C	35.792	-305.122	-364.993
O	36.203	-304.892	-363.651
C	35.073	-304.655	-362.831
H	36.923	-303.382	-360.484
H	38.477	-302.771	-359.868
H	37.937	-304.957	-358.85
H	39.338	-305.142	-359.935
H	36.69	-307.67	-360.787
H	37.221	-307.23	-359.159
H	38.694	-308.981	-360.38
H	39.553	-307.562	-359.741
H	40.379	-308.979	-362.709
H	41.155	-308.177	-361.339
H	42.011	-307.321	-363.602
H	41.416	-306.109	-362.465
H	39.583	-308.214	-364.907
H	40.941	-307.379	-365.703

H	38.796	-307.073	-366.977
H	39.455	-305.547	-366.346
H	36.726	-305.509	-366.886
H	37.561	-304.328	-365.854
H	35.167	-306.033	-365.056
H	35.178	-304.283	-365.373
H	35.431	-304.529	-361.799
H	34.36	-305.497	-362.851
H	34.532	-303.735	-363.119
C	30.378	-299.643	-368.628
O	30.426	-301.031	-368.928
C	29.296	-301.403	-369.706
C	29.365	-302.858	-370.048
O	30.459	-303.202	-370.904
C	30.199	-303.02	-372.303
C	29.394	-304.123	-372.951
O	28.018	-303.752	-372.955
C	27.086	-304.745	-373.389
C	26.756	-305.795	-372.36
O	26.342	-305.284	-371.092
C	24.974	-304.905	-370.99
C	24.825	-304.093	-369.735
O	25.54	-302.853	-369.745
C	24.823	-301.814	-370.404
C	25.699	-300.603	-370.525
O	26.922	-300.811	-371.227
C	26.714	-300.883	-372.627
H	29.252	-300.806	-370.633
H	28.359	-301.217	-369.148
H	28.403	-303.204	-370.467
H	29.529	-303.419	-369.116
H	31.199	-302.946	-372.754
H	29.695	-302.061	-372.502
H	29.571	-305.056	-372.391
H	29.739	-304.301	-373.985
H	27.424	-305.238	-374.318
H	26.176	-304.18	-373.641
H	27.662	-306.382	-372.142
H	26.009	-306.497	-372.774
H	24.333	-305.803	-370.939
H	24.646	-304.319	-371.864
H	25.267	-304.679	-368.913
H	23.764	-303.936	-369.477
H	23.915	-301.55	-369.832
H	24.488	-302.134	-371.407
H	25.992	-300.29	-369.511
H	25.136	-299.759	-370.962
H	27.702	-300.962	-373.102
H	26.213	-299.983	-373.025
H	26.12	-301.769	-372.911
C	33.758	-300.578	-368.525
H	33.659	-301.334	-367.729
H	33.165	-300.935	-369.383
C	35.233	-300.418	-368.915
C	35.987	-301.725	-369.198
H	35.311	-299.749	-369.789
H	35.759	-299.906	-368.09
H	35.802	-302.422	-368.362
H	35.595	-302.207	-370.11
C	37.5	-301.5	-369.325
S	38.399	-303.072	-369.406
H	37.854	-300.93	-368.45
H	37.731	-300.898	-370.22
C	40.171	-302.607	-369.31
C	40.501	-301.88	-368.017
H	40.028	-302.399	-367.168
H	40.096	-300.855	-368.028
S	42.293	-301.784	-367.711
C	42.394	-301.797	-365.901
H	41.64	-301.108	-365.495
H	43.384	-301.381	-365.643
C	42.276	-303.214	-365.331
C	42.598	-303.303	-363.828
H	43	-303.849	-365.874
H	41.276	-303.632	-365.537
H	43.393	-302.573	-363.59
H	43.037	-304.293	-363.614
C	41.415	-303.084	-362.87
S	40.589	-301.484	-363.08
H	41.776	-303.13	-361.829
H	40.682	-303.895	-362.989
C	38.969	-301.99	-363.722
C	38.394	-300.86	-364.548
H	39.107	-302.873	-364.368
H	39.096	-300.559	-365.344
H	38.252	-299.972	-363.909
S	36.812	-301.268	-365.348
C	35.91	-299.716	-365.066
H	35.058	-299.674	-365.764
H	36.578	-298.874	-365.315
C	35.399	-299.606	-363.629
C	34.822	-298.224	-363.293
H	36.212	-299.834	-362.92
H	34.621	-300.374	-363.477
H	35.63	-297.472	-363.32
H	34.107	-297.937	-364.084
C	34.11	-298.175	-361.93
S	35.204	-298.526	-360.518
H	33.273	-298.889	-361.953
H	33.669	-297.177	-361.77
C	34.079	-299.237	-359.265
C	33.908	-300.788	-359.407
H	34.615	-299.078	-358.312
H	33.307	-301.183	-358.571
H	34.909	-301.243	-359.31
S	33.207	-301.425	-360.966
C	31.432	-301.397	-360.585
H	31.133	-300.376	-360.3
H	31.263	-302.036	-359.702
C	30.571	-301.882	-361.762
C	30.594	-300.929	-362.963
H	29.523	-301.995	-361.43
H	30.904	-302.887	-362.073
H	31.633	-300.781	-363.302
H	30.223	-299.941	-362.635
C	29.74	-301.424	-364.129
S	29.611	-300.207	-365.467
H	30.126	-302.377	-364.526
H	28.71	-301.608	-363.78
C	31.252	-300.274	-366.255

C	31.371	-299.296	-367.411	H	37.966	-301.505	-361.833
H	32.009	-300.045	-365.487	H	37.002	-302.578	-362.841
H	31.085	-298.296	-367.036	H	41.788	-302.186	-370.78
S	33.104	-298.993	-367.93	H	40.197	-302.223	-371.55
H	30.618	-299.035	-369.52	H	40.652	-303.6	-369.264
H	29.355	-299.353	-368.323	H	31.446	-301.303	-366.6
H	32.988	-297.364	-358.859	Na	38.042	-306.021	-362.971
H	32.165	-298.366	-360.075				

Table S5. XYZ coordinates for structure $[SL_2]^+$ shown in Figure S4.

C	-400.453	467.782	237.469	C	-391.625	464.615	233.205	H	-391.567	462.843	241.169
O	-400.909	467.38	236.176	C	-392.829	465.473	233.462	H	-390.698	461.7	238.45
C	-400.591	466.023	235.876	O	-393.98	464.801	233.968	H	-390.521	463.41	238.866
C	-400.712	465.825	234.405	C	-393.832	464.356	235.303	H	-392.421	465.17	238.537
O	-399.9	466.82	233.779	C	-398.444	468.556	238.892	H	-392.787	464.584	240.172
C	-399.769	466.692	232.355	C	-398.92	468.184	237.472	H	-395.174	464.322	239.624
C	-400.125	467.987	231.685	S	-398.675	469.523	236.259	H	-394.876	464.681	237.921
O	-401.321	468.568	232.204	C	-397.003	469.217	235.619	H	-394.177	467.015	238.522
C	-402.518	467.925	231.78	C	-396.96	468.006	234.69	H	-394.539	466.682	240.225
C	-403.617	468.46	232.629	C	-395.604	467.794	234	H	-396.925	466.195	239.638
O	-403.215	468.352	233.995	C	-395.148	468.964	233.112	H	-396.557	466.445	237.917
C	-404.222	468.871	234.865	S	-396.363	469.399	231.832	H	-388.877	461.509	240.427
C	-403.57	469.299	236.143	H	-400.654	467.001	238.224	H	-389.374	461.875	242.098
O	-402.36	470.02	235.901	H	-401.061	468.655	237.755	H	-387.234	463.171	240.051
C	-402.555	471.39	235.559	H	-401.251	465.338	236.44	H	-387.258	463.285	241.828
C	-401.266	471.907	235.02	H	-399.552	465.781	236.168	H	-386.258	465.283	240.767
O	-400.901	471.08	233.921	H	-401.766	465.928	234.09	H	-387.728	465.719	241.656
C	-399.739	471.577	233.283	H	-400.389	464.806	234.121	H	-386.735	467.388	239.584
S	-393.644	457.41	245.375	H	-400.401	465.876	231.97	H	-388.016	467.269	238.351
C	-394.901	458.526	244.686	H	-398.727	466.41	232.129	H	-385.537	465.523	238.454
C	-394.275	459.797	244.085	H	-400.167	467.866	230.588	H	-385.713	466.94	237.398
C	-393.443	459.542	242.822	H	-399.337	468.729	231.887	H	-385.749	463.66	237.099
C	-392.585	460.744	242.433	H	-402.461	466.829	231.912	H	-385.173	464.898	235.957
S	-391.68	460.449	240.891	H	-402.706	468.108	230.705	H	-386.001	462.978	234.759
C	-391.005	462.109	240.569	H	-404.566	467.92	232.451	H	-387.019	464.386	234.43
C	-391.114	462.505	239.079	H	-403.795	469.52	232.363	H	-389.065	464.479	236.084
S	-392.824	462.808	238.535	H	-405.006	468.112	235.042	H	-389.745	462.858	236.286
C	-393.086	464.506	239.114	H	-404.725	469.741	234.407	H	-389.983	462.592	233.866
C	-394.546	464.925	238.945	H	-403.273	468.409	236.719	H	-389.092	464.103	233.577
C	-394.817	466.413	239.191	H	-404.266	469.871	236.783	H	-391.934	463.682	232.699
C	-396.285	466.757	238.938	H	-402.899	471.969	236.436	H	-390.941	465.149	232.519
S	-396.638	468.529	239.119	H	-403.326	471.504	234.775	H	-393.169	465.929	232.519
C	-389.492	462.189	241.045	H	-400.488	471.878	235.807	H	-392.524	466.302	234.126
O	-389.103	463.544	240.883	H	-401.374	472.963	234.706	H	-393.548	465.172	235.988
C	-387.692	463.708	240.904	H	-399.882	472.601	232.894	H	-393.088	463.545	235.394
C	-387.353	465.158	240.783	H	-399.522	470.918	232.43	H	-394.808	463.958	235.614
O	-387.957	465.674	239.594	H	-398.861	471.579	233.954	H	-398.808	469.561	239.163
C	-387.245	466.697	238.89	H	-394.444	456.496	245.9	H	-398.869	467.858	239.633
C	-386.273	466.132	237.901	H	-395.599	458.795	245.497	H	-398.359	467.301	237.132
O	-386.965	465.345	236.932	H	-395.482	457.976	243.928	H	-396.281	469.107	236.443
C	-386.079	464.397	236.344	H	-393.63	460.255	244.858	H	-396.723	470.128	235.064
C	-386.733	463.665	235.213	H	-395.055	460.541	243.85	H	-397.737	468.121	233.917
O	-387.835	462.832	235.596	H	-394.12	459.284	241.988	H	-397.217	467.094	235.255
C	-389.116	463.476	235.627	H	-392.781	458.672	242.975	H	-395.658	466.883	233.378
C	-389.78	463.598	234.278	H	-391.856	460.965	243.232	H	-394.84	467.588	234.771
O	-390.975	464.348	234.438	H	-393.222	461.634	242.301	H	-394.2	468.702	232.612

H	-394.949	469.86	233.722	H	-395.654	470.318	231.199	Na	-400.998	468.854	234.463

Table S6. XYZ coordinates for structure $[SL_4]^+$ shown in Figure S4.

C	443.946	-162.948	-755.373	C	432.589	-170.267	-758.201
O	444.777	-163.78	-754.575	O	433.41	-169.48	-759.07
C	444.184	-163.991	-753.297	C	432.673	-168.888	-760.142
C	445.029	-164.95	-752.524	C	433.481	-167.77	-760.709
O	445.577	-165.891	-753.439	O	433.496	-166.708	-759.757
C	446.216	-166.968	-752.769	C	434.599	-165.818	-759.901
C	446.889	-167.825	-753.786	C	434.284	-164.501	-759.256
O	447.951	-167.087	-754.388	O	433.435	-163.663	-760.035
C	448.664	-167.847	-755.354	C	432.059	-163.993	-759.958
C	449.363	-166.877	-756.242	H	431.65	-166.566	-752.623
O	448.34	-166.04	-756.78	H	433.169	-166.408	-751.73
C	448.798	-165.11	-757.767	H	433.678	-167.685	-753.68
C	449.328	-163.864	-757.135	H	434.347	-166.056	-753.932
O	448.39	-163.51	-756.128	H	434.109	-165.934	-756.352
C	448.84	-162.473	-755.266	H	432.587	-166.535	-757.023
C	447.843	-162.372	-754.159	H	434.814	-168.154	-755.71
O	447.649	-163.618	-753.484	H	434.371	-168.13	-757.416
C	448.631	-163.844	-752.485	H	431.535	-168.61	-757.328
H	443.168	-164.41	-753.417	H	431.402	-170.134	-756.434
H	444.061	-163.035	-752.755	H	431.741	-170.712	-758.747
H	444.426	-165.452	-751.744	H	433.212	-171.102	-757.849
H	445.837	-164.422	-751.987	H	431.711	-168.473	-759.793
H	445.477	-167.55	-752.186	H	432.44	-169.654	-760.903
H	446.977	-166.606	-752.054	H	433.064	-167.408	-761.666
H	446.141	-168.126	-754.544	H	434.498	-168.149	-760.914
H	447.261	-168.753	-753.317	H	434.836	-165.63	-760.963
H	447.972	-168.447	-755.976	H	435.495	-166.269	-759.437
H	449.354	-168.553	-754.858	H	435.207	-163.916	-759.125
H	449.926	-167.392	-757.042	H	433.874	-164.674	-758.248
H	450.091	-166.288	-755.653	H	431.521	-163.231	-760.542
H	447.907	-164.864	-758.368	H	431.684	-163.971	-758.92
H	449.525	-165.571	-758.457	H	431.836	-164.981	-760.395
H	449.45	-163.051	-757.875	C	439.093	-164.781	-751.81
H	450.326	-164.054	-756.698	O	440.063	-163.848	-751.346
H	448.928	-161.513	-755.808	C	439.936	-163.492	-749.965
H	449.838	-162.7	-754.847	C	440.63	-164.424	-749.018
H	446.866	-162.116	-754.603	O	439.961	-165.676	-749.07
H	448.095	-161.561	-753.452	C	440.552	-166.74	-748.326
H	449.654	-163.864	-752.895	C	440.222	-166.765	-746.865
H	448.426	-164.828	-752.04	O	440.931	-165.728	-746.204
H	448.589	-163.084	-751.684	C	440.856	-165.844	-744.791
C	431.416	-164.109	-753.164	C	441.634	-164.722	-744.163
O	432.589	-164.681	-752.603	O	441.841	-165.053	-742.801
C	432.653	-166.109	-752.654	C	442.543	-164.065	-742.057
C	433.405	-166.626	-753.843	C	442.997	-164.69	-740.772
O	432.604	-166.494	-755.011	O	444.173	-165.452	-741.033
C	433.326	-166.703	-756.223	C	444.436	-166.403	-740.01
C	433.958	-168.058	-756.395	C	445.753	-167.064	-740.258
O	433.123	-169.169	-756.069	O	445.707	-167.741	-741.506
C	432.098	-169.502	-757.005	C	446.889	-168.48	-741.739

H	438.875	-163.408	-749.669
H	440.368	-162.483	-749.888
H	440.606	-164.005	-747.995
H	441.691	-164.521	-749.31
H	441.651	-166.755	-748.437
H	440.171	-167.656	-748.805
H	440.504	-167.754	-746.461
H	439.132	-166.652	-746.724
H	441.301	-166.795	-744.442
H	439.809	-165.824	-744.435
H	441.094	-163.766	-744.282
H	442.598	-164.627	-744.696
H	441.894	-163.188	-741.885
H	443.439	-163.707	-742.596
H	442.176	-165.326	-740.397
H	443.205	-163.935	-739.993
H	443.648	-167.178	-739.999
H	444.436	-165.919	-739.017
H	445.972	-167.776	-739.442
H	446.552	-166.3	-740.242
H	447.069	-169.231	-740.949
H	447.778	-167.83	-741.822
H	446.755	-169.005	-742.696
C	438.578	-161.601	-742.901
O	438.444	-162.958	-743.293
C	437.451	-163.726	-742.615
C	436.056	-163.485	-743.124
O	435.467	-162.403	-742.409
C	434.447	-161.696	-743.111
C	433.218	-162.481	-743.499
O	433.367	-163.425	-744.563
C	433.568	-162.81	-745.832
C	433.495	-163.827	-746.933
O	434.694	-164.578	-747.123
C	434.798	-165.702	-746.256
C	435.974	-166.523	-746.68
O	435.821	-167.055	-748.002
C	436.438	-166.285	-749.033
C	435.845	-166.709	-750.362
O	436.821	-166.864	-751.393
C	437.338	-168.182	-751.48
H	437.47	-163.557	-741.525
H	437.749	-164.773	-742.78
H	435.426	-164.383	-743.007
H	436.122	-163.273	-744.203
H	434.866	-161.188	-743.999
H	434.134	-160.903	-742.412
H	432.402	-161.769	-743.724
H	432.892	-163.07	-742.629
H	434.561	-162.33	-745.881
H	432.812	-162.026	-746.013
H	433.324	-163.323	-747.895
H	432.621	-164.48	-746.764
H	433.891	-166.331	-746.311
H	434.913	-165.38	-745.207
H	436.091	-167.396	-746.02
H	436.896	-165.924	-746.59
H	437.522	-166.482	-749.042
H	436.317	-165.202	-748.861
H	435.128	-165.963	-750.739

H	435.273	-167.638	-750.209
H	437.846	-168.494	-750.552
H	438.068	-168.183	-752.302
H	436.547	-168.917	-751.713
H	443.147	-159.734	-762.186
S	443.942	-158.684	-762.318
C	443.935	-158.17	-760.58
H	442.883	-158.009	-760.294
H	444.452	-157.203	-760.474
C	444.581	-159.233	-759.695
C	444.29	-159.066	-758.196
H	445.672	-159.245	-759.867
H	444.211	-160.221	-760.021
H	443.213	-158.862	-758.051
H	444.825	-158.179	-757.815
C	444.659	-160.318	-757.384
S	443.658	-161.756	-757.869
H	444.511	-160.129	-756.31
H	445.725	-160.56	-757.529
C	444.4	-163.085	-756.887
C	444.034	-164.416	-757.53
H	444.418	-164.438	-758.564
H	442.937	-164.506	-757.606
S	444.643	-165.922	-756.7
C	443.134	-166.349	-755.769
H	442.638	-165.402	-755.509
H	443.414	-166.842	-754.825
C	442.179	-167.244	-756.566
C	440.695	-167.166	-756.154
H	442.529	-168.288	-756.493
H	442.232	-166.978	-757.637
H	440.138	-167.891	-756.774
H	440.301	-166.177	-756.436
C	440.371	-167.427	-754.667
S	440.877	-166.107	-753.516
H	440.886	-168.343	-754.336
H	439.291	-167.614	-754.549
C	439.388	-165.054	-753.354
C	439.527	-163.688	-754.063
H	438.524	-165.594	-753.777
H	440.534	-163.275	-753.895
H	438.821	-162.965	-753.622
S	439.228	-163.735	-755.855
C	437.7	-162.759	-756.006
H	437.516	-162.605	-757.081
H	437.874	-161.764	-755.563
C	436.477	-163.416	-755.364
C	435.22	-162.541	-755.465
H	436.682	-163.617	-754.298
H	436.285	-164.393	-755.841
H	434.934	-162.412	-756.524
H	435.454	-161.533	-755.078
C	434.035	-163.101	-754.678
S	432.604	-161.981	-754.691
H	434.357	-163.293	-753.644
H	433.723	-164.067	-755.1
C	431.588	-162.519	-753.258
C	431.995	-161.832	-751.898
H	430.594	-162.116	-753.523
H	431.355	-162.218	-751.089

H	431.789	-160.753	-751.98
S	433.729	-162.035	-751.39
C	433.847	-161.085	-749.849
H	433.061	-161.417	-749.151
H	433.662	-160.017	-750.053
C	435.235	-161.303	-749.242
C	435.555	-160.553	-747.941
H	436.004	-161.038	-749.992
H	435.357	-162.385	-749.052
H	434.73	-160.677	-747.219
H	435.629	-159.47	-748.139
C	436.873	-161.062	-747.34
S	437.288	-160.343	-745.722
H	437.702	-160.848	-748.034
H	436.828	-162.157	-747.214
C	438.925	-161.11	-745.439
C	439.539	-160.984	-744.007
H	438.818	-162.18	-745.681
H	440.442	-161.621	-743.993
S	440.23	-159.346	-743.587
C	438.813	-158.267	-743.277
H	438.618	-158.291	-742.193
H	437.896	-158.625	-743.768
C	439.116	-156.832	-743.732
C	439.181	-156.65	-745.255
H	440.071	-156.504	-743.283
H	438.345	-156.146	-743.339
H	438.189	-156.887	-745.678
H	439.904	-157.353	-745.707
C	439.55	-155.212	-745.619
S	439.583	-154.941	-747.41
H	440.537	-154.949	-745.204
H	438.816	-154.515	-745.18
H	439.809	-153.637	-747.38
H	439.016	-161.524	-741.889
H	437.61	-161.07	-742.887
H	430.545	-164.356	-752.531
H	431.2	-164.515	-754.169
H	438.073	-164.367	-751.697
H	439.117	-165.723	-751.236
H	444.021	-161.903	-755.019
H	442.88	-163.229	-755.294
H	445.497	-162.965	-756.915
H	439.625	-160.694	-746.182
Na	446.791	-165.02	-755.172

References

1. Wesdemiotis, C.; Solak, N.; Polce, M.J.; Dabney, D.E.; Chaicharoen, K.; Katzenmeyer, B.C. Fragmentation pathways of polymer ions. *Mass Spectrom. Rev.* **2011**, *30* (4), 523–559. https://doi.org/10.1002/MAS.20282

2. Williams-Pavlatos, K. Multidimensional Mass Spectrometry Analysis and Imaging of Macromolecules and Material Surfaces. Ph.D. Dissertation, The University of Akron, May 2023. http://rave.ohiolink.edu/etdc/view?acc_num=akron1682339700569807

3. Bush, M.F.; Campuzano, I.D.G.; Robinson, C.V. Ion mobility mass spectrometry of peptide ions: Effects of drift gas and calibration strategies. *Anal. Chem.* **2012**, *84* (16), 7124–7130. https://doi.org/10.1021/AC3014498

4. Geue, N.; Bennett, T.S.; Arama, A.-A.-M.; Ramakers, A.I.; Whitehead, G.F.S.; Timco, G.A.; Armentrout, P.B.; McInnes, E.J.; Burton, N.A.; Winpenny, R.E.P.; Barran, P.E. Disassembly mechanisms and energetics of polymetallic rings and rotaxanes. *J. Am. Chem. Soc.* **2022**, *144* (49) 22528–22539. https://doi.org/10.1021/jacs.2c07522

5. Geue, N.; Winpenny, R.E.P.; Barran, P.E. Ion mobility mass spectrometry for large Synthetic molecules: Expanding the analytical toolbox. *J. Am. Chem. Soc.* **2024**, 46 (13), 8800–8819. https://doi.org/10.1021/jacs.4c00354
